# Using Biobrane: Techniques to Make Life Easier

**Published:** 2010-12-20

**Authors:** Nicholas S. Solanki, Katarzyna M. Nowak, Ian P. Mackie, John E. Greenwood

**Affiliations:** Burns Unit, Royal Adelaide Hospital, North Terrace, Adelaide 5000, South Australia, Australia

## Abstract

**Aims:** To facilitate the use of Biobrane for those burn care practitioners not familiar with this material. **Methods:** Two techniques have been developed through extensive use of Biobrane over many years, in both sheet and glove form. These techniques have been described and illustrated with photographs. **Results:** The use of these techniques has allowed the corresponding author to markedly reduce operating time and to easily apply the material single-handedly. **Conclusion:** Biobrane is a biosynthetic skin substitute primarily designed for the definitive treatment of superficial partial-thickness to mid-dermal burn injury. Once experienced with its use, the material is quite ubiquitous. The described techniques will facilitate the use of Biobrane for those not familiar with it.

Biobrane is a biosynthetic dressing which was first developed by Aubrey Woodroof in 1979, is now manufactured by Bertek Pharmaceuticals Inc, Sugarland, Texas, and distributed by Smith & Nephew Medical Ltd, Hull, UK. It has a bilayer structure consisting of silicone bonded to woven nylon containing peptides derived from type I porcine collagen. Small pores allow drainage of fluid from the wound. It has found widespread use in the treatment of partial-thickness burns and has been shown to decrease healing time, analgesic requirements, and the length of inpatient stay when compared to traditional dressings.^1^^-^3 Two issues have been addressed and solved during extensive use of Biobrane in our practice.^4^ With the development and introduction of newer materials, such as AWBAT-plus, it is important that techniques to facilitate their application be shared.

It is generally agreed that Biobrane should be stretched and secured under tension. This has been reported with fixation by staples or steri-strips.^5^ In a study of UK burn units, 4 main materials were commonly used to secure Biobrane; staples, glue, steri-strips, and bandages.^6^ Surgical staples are a fast and secure method for applying Biobrane but require later removal, which is uncomfortable in the ward environment. Glue provides secure fixation but is expensive and associated with a prolonged application time (allowing the glue to set before the Biobrane can be stretched across the wound). Glue can also be difficult to apply to the underside of a patient due to the tendency for it to “drip” downwards under the influence of gravity and, like staples, it can make Biobrane more difficult and painful to remove (especially if the glue has not degraded). Steri-strips and bandages are less commonly used techniques, possibly due to expense and the time-consuming nature of applying steri-strips. There is an inherent instability when fixation is by bandages alone.

## TECHNIQUE 1

**Figure 1:**
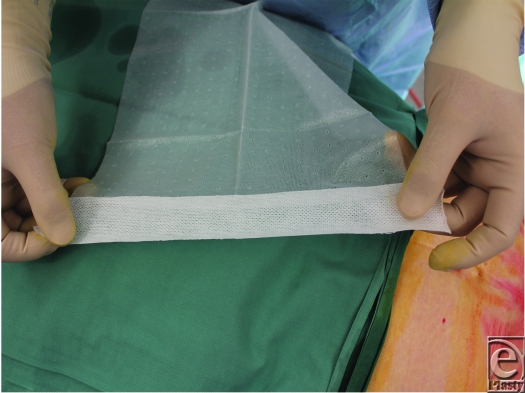
Prior to application, the free edge of the Biobrane is “picked up” by a strip of Hypafix.

**Figure 2:**
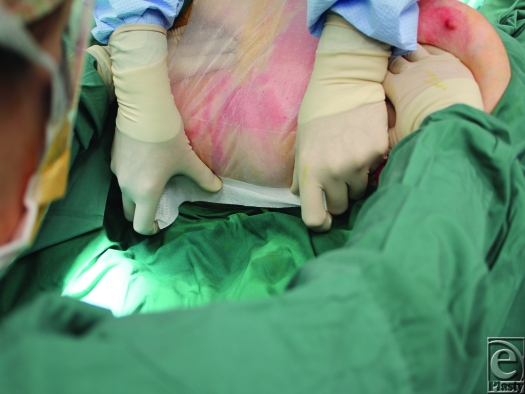
This facilitates single user application to, for example, the posterior flanks.

**Figure 3:**
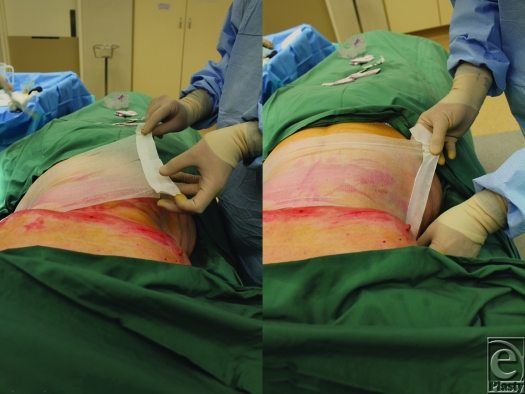
(*a*) and (*b*) The opposite edge can be similarly placed under tension prior to adhesion by the tape.

This has successfully been used in our burns unit for many years. The wound bed is meticulously cleaned and prepared according to the guidelines developed in the consensus meeting^5^ and reiterated in our previous work^4^ and then Tincture of Benzoin (Friar's Balsam) is applied to the clean skin around the wound edge to make it “tacky.” The sheet of Hypafix (BSN Medical GmBH, Hamburg, Germany) is folded and then cut to form ribbons approximately 3- to 4 cm wide. A sheet of Biobrane is applied to the wound bed and a wide strip of Hypafix is first cut to size and then stuck to one side of the Biobrane (Fig 1) and then used to anchor it to the normal skin surrounding the burn wound edge (Fig 2). The Biobrane is then stretched across the wound by pulling against the anchored edge and trimmed to size. The free edge of Biobrane opposite the fixed edge is “picked up” with a strip of Hypafix, ensuring that half of the adhesive surface of Hypafix is still free and is then used to pull the Biobrane over the wound (Figs 3a and 3b). The free edge of the Hypafix is then secured to the intact skin beyond the burn wound. We recommend that the Hypafix be not applied to the burn wound itself, as exudate from this area makes adherence difficult. The remaining 2 edges are then similarly stretched, trimmed, and secured with Hypafix.

This technique allows rapid application of Biobrane to a wound in any position which can be performed by a single operator. The Hypafix tape used is inexpensive, provides secure fixation, and does not cause discomfort to the patient upon removal. The adhesive qualities of Hypafix also do not seem to degrade with sterilization. We recommend this technique as a fast and reliable method of applying Biobrane to burn wounds.

## TECHNIQUE 2

Passive flexion of the wrist on the operating table produces extension of the metacarpophalangeal joints of the hand via the “passive” tenodesis release effect.

In burns surgery, Biobrane gloves are used within the first 24 hours to cover clean partial-thickness burn wounds of the hand. The glove promotes early movement of the burned hand and helps create a barrier from infection. However, the insertion of the fingers into the thin adherent material of the Biobrane glove can create difficulty for the single surgeon. Described below is the application of the tenodesis release to produce passive finger extension and allow single-handed application of the Biobrane glove for the anesthetized patient.

The tenodesis effect is based on the principle of developing and using passive tension in a 2 (or more) joint contractile structure by moving one of the joints to produce movement at another of the joints. It has been used to substitute for lost function (creating a functional grip in paralysis), in tenodesis surgery of the hand, in the use of dynamic hand splints, to test for integrity of the extensor tendons of the hand (tenodesis test), and as the foundation of self-defence moves to disenable grasp of a weapon.

**Figure 4:**
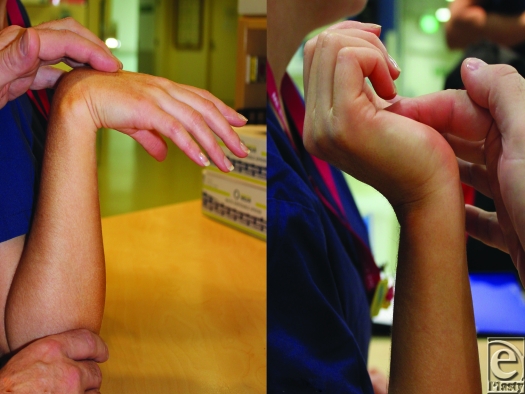
(*a*) Tenodesis “release” with wrist flexion and (*b*) tenodesis “grasp” with wrist extension.

**Figure 5:**
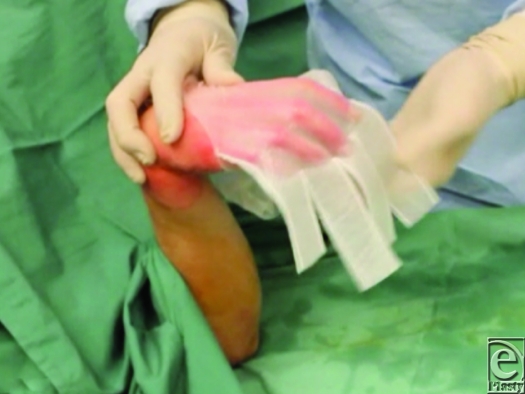
Using tenodesis release to aid application of a Biobrane glove.

Active wrist flexion stretches the extensor digitorum communis. The tension created pulls the fingers into extension (Fig 4a). A similar but converse action results in the tenodesis grasp effect, in which wrist extension results in finger flexion (Fig 4b). With the application of the tenodesis effect during surgery, finger extension is maintained without individual manipulation of the digits, leaving one hand completely free to apply the glove to the burned hand (Fig 5). Once the digits are in their respective glove fingers, traction on the axial seams allows smooth apposition all the way down to the Web spaces. At this point, traction on the “gauntlet” part of the glove (the wrist and forearm component) causes further smooth apposition, and Hypafix tapes can be used to close the open forearm seam and fix the proximal end in place.

## CONCLUSION

Extensive use of any technology encourages the development of “short-cuts” and manoeuvres designed to facilitate, and thus expedite, procedures. Although surgeons may arrive at these techniques spontaneously with time, it behoves us to share information that makes all of our surgical lives easier.
